# Management of Obstetric Perineal Tears: Do Obstetrics and Gynaecology Residents Receive Adequate Training? Results of an Anonymous Survey

**DOI:** 10.1155/2012/316983

**Published:** 2011-07-24

**Authors:** A. Cornet, O. Porta, L. Piñeiro, E. Ferriols, I. Gich, J. Calaf

**Affiliations:** ^1^Department of Obstetrics and Gynaecology, Viladecans Hospital, Avenue Gavà 38 Viladecans, 08840 Barcelona, Spain; ^2^Department of Obstetrics and Gynaecology, Hospital of the Hdy cross and Saint Paul, Mas Casanovas 90, 08025 Barcelona, Spain; ^3^Department of Epidemiology, Hospital of the Hdy cross and Saint Paul, Mas Casanovas 90, 08025 Barcelona, Spain

## Abstract

*Background/Aim*. To evaluate the obstetrics and gynaecology residents' perspective of their training and experience in the management of perineal tears that occur during assisted vaginal delivery. We hypothesised that residents would perceive room for improvement in their knowledge of pelvic floor anatomy and the training received in tears repair. *Design*. Descriptive cross-sectional study. *Population/Setting*. Seventy-two major residents from all teaching hospitals in Catalonia. *Methods*. A questionnaire was designed to evaluate experience, perception of the training and supervision provided. *Results*. The questionnaire was sent to all residents (*n* = 72), receiving 46 responses (64%). The participants represented 15 out of the 16 teaching hospitals included in the study (94% of the hospitals represented). Approximately, 52% of residents were in their third year while 48% were in their fourth. The majority of them thought that their knowledge of pelvic floor anatomy was poor (62%), although 98% felt confident that they would know when an episiotomy was correctly indicated. The survey found that they lacked experience in the repair of major degree tears (70% had repaired fewer than ten), and most did not carry out followup procedures. *Conclusion*. The majority of them indicated that more training in this specific area is necessary (98%).

## 1. Introduction

Obstetric anal sphincter injury (OASI) is said to occur in approximately 1–4% of all deliveries, although the true incidence may be substantially higher [1]. Followup has shown anal incontinence (AI) symptoms in up to 57% of those who undergo primary repair [2]. Longterm followup of such symptomatic patients shows a high prevalence of women with AI after OASI [3]. Women with OASI are twice as at risk of suffering AI six-month postpartum [4]. However, about 60–80% of women who suffer an obstetric anal sphincter injury but have a good external anal sphincter (EAS) repair remain asymptomatic at twelve months. Most women who remain symptomatic describe incontinence of flatus or faecal urgency [5]. Although AI is not a life-threatening condition, it may affect women psychologically and physically [6, 7].

There is no other moment in a woman's life when pelvic floor structures are more vulnerable than during childbirth. DeLancey et al. [8] showed the association between delivery and injuries caused to the levator ani muscles, and Hendrix et al. [9] has ascertained the increase in the risk of pelvic organ prolapse after vaginal delivery. But the strongest data suggesting a causal relationship between childbirth and levator trauma is provided by ultrasound studies comparing pelvic floor structures before and after childbirth [10, 11]. If professionals practising in the labour ward are made more aware of the risk to pelvic floor structures during delivery, they are more likely to adopt the appropriate preventive measures. 

Various studies suggest that the degree of knowledge of obstetrics and gynaecology specialists in the repair of perineal injuries, specifically those involving the anal sphincter, is limited. According to Fernando et al. [12] and McLennan et al. [13], the training given to obstetrics and gynaecology residents on this subject is insufficient. Furthermore, these studies show the positive effect of introducing specific training actions [14–17]. Evaluating this situation for gynaecology residents and future specialists is of great relevance, since measures can be taken during their training period which aim to maximise aptitudes and, thereby, allow residents to undertake a more comprehensive and safer professional practice. At this point in time, no specific formal training regarding OASI management is contemplated in the educational programme of residents in Catalonia.

We hypothesised that there was room for improvement in the knowledge of pelvic floor anatomy for obstetrics and gynaecology residents in Catalonia (a region in the north-east of Spain with approximately eight million inhabitants), and that residents had poor experience in repairing third and fourth degree perineal tears, and insufficient knowledge of the risk that their obstetric manoeuvres entailed for the female pelvic floor. 

The aim of this research was to verify the validity of this hypothesis through a questionnaire explicitly designed for this purpose. If shown to be valid, the hypothesis will reveal the need for specific training actions to be formally implemented.

## 2. Material and Methods

A descriptive cross-sectional study was designed. The population included in the sample consisted of third and fourth year obstetrics and gynaecology residents in Catalonia. First and second year residents were not included, as complex tears are usually repaired by experienced residents (i.e., third and fourth year residents). A questionnaire was designed containing 40 items, which was reviewed by three senior doctors and pelvic floor pathology experts that had no connection with our centre—although this was not formally validated. The questionnaire was divided into five blocks: affiliation, anatomy, episiotomy, perineal tears, and teaching (Table 4). 

The study took place between October 2007 and January 2008. We sent out 72 questionnaires by e-mail to all residents in their third and fourth year with the endorsement of the *Acadèmia de Ciències Mèdiques de Catalunya i Balears*. Emphasis was also placed on information received through our contact with residents and associate physicians from the hospitals involved. The number of residents per year differs from one hospital to the next, with as many as seven residents in the bigger hospitals to one resident in the smaller ones. There are 16 hospitals with residency programmes in Catalonia, and all of them were included. The sample was constituted by 12-male residents and 60-female residents (there are more female than male students at medical schools and, consequently, there are more female than male residents in several specialties). Participants remained anonymous—although mention was made of the hospital of origin—and all responses were treated confidentially. A Microsoft Excel database was created with all the variables as dichotomous except those of the affiliation and variables 18 and 19 (ordinal from 0 to 5) and 20 (ordinal with three possibilities). 

Three variables from the original questionnaire were modified for the statistical analysis. First, a new dichotomous variable denominated “deliveries per resident” (<1000 deliveries/resident, >1000 deliveries/resident) was created in the affiliation block, calculated according to the discrete quantitative variable of “number of deliveries/year” for each hospital and the discrete quantitative variable of “number of residents per year in that hospital.” Also, the average quantitative variable of “number of residents/year” of that first block was converted into a dichotomous variable (≤2 residents/year, >2 residents/year). Finally, in the tears block, the discrete quantitative variable 20 was converted into an ordinal variable (Table 4). 

Frequency tables were calculated for each variable, with confidence intervals of 95% for some of the variables of each block which were considered outstanding and representative. In addition, aggregate index rates were calculated for each block. Finally, cross-data from Student's *t* test was used to compare each rate on the basis of other variables, facilitating the standard and mean deviation. All analyses were done using the SPSS statistical package (V15.0). 

The variables that were considered representative of each block were as follows.

**Anatomy**

Question 6 was selected as representative of the block since it evaluated each resident's subjective perception of his/her knowledge of pelvic floor anatomy.

**Episiotomy**

Questions 12 and 15 could reflect that a restrictive policy for episiotomy may promote the learning of its indications.

**Tears**

Questions 18 and 19 would be useful in comparing how self-confident the residents felt when repairing tears versus major surgery (caesarean section). Complementing this question, question 20 reflects the frequency in which residents were confronted with these kinds of tear. As explained above, this variable was converted into a dichotomous variable to facilitate the statistical analysis. Questions 21, 22, and 23 were useful to analyse how well residents knew the definition of major tears, but we considered questions 18 and 19 more relevant for showing how insecure they felt when it came to repairing them.

**Teaching**

Questions 25 and 26 were selected because they reflected the morbidity associated with perineal tears.

Questions 24, 31, and 33 were the most representative in terms of the evaluation of teaching.

Participants were not asked to provide informed consent, and they voluntarily participated after having received written information regarding the purposes of the study. No compensation was provided for completing the survey. The study was revised and approved by the Institutional Ethics Committee.

## 3. Results and Discussion

### 3.1. Results

We received 46 questionnaires out of the 72 (64%) sent out to all third and fourth year residents, representing 15 out of the 16 teaching hospitals in Catalonia participating in the study (94% of hospitals). There were 24 (52%) in their third year while the remaining 22 (48%) were in their fourth. There were 37 (80%) females against 9 (20%) males (Table 1). Aggregate index rates calculated for each block are shown in Table 2.

No significant differences were found in the comparison carried out between the variables of each block when crossed with the affiliation variables. We found no systematic differences between the mean scores of each block, depending on year of residence, level of hospital, number of residents per hospital, or number of deliveries per resident (Table 3).

About 28 (62%) answered that their knowledge of pelvic floor anatomy was inadequate (95% CI: 45.4–74.9). Moreover, 45 (98%) of respondents thought they knew when an episiotomy was indicated (95% CI: 88.5–99.9). In 37 (80%) of the centres a restrictive policy for episiotomy was used (95% CI: 66.1–90.6).

Of the Catalan residents, 32 (70%) had repaired less than 10 third or fourth degree perineal tears (95% CI: 54.2–82.3). We observed significant differences with regard to how residents graded their self-confidence in the execution of a caesarean section and in the repair of a third or fourth degree tear. Residents were asked to grade their level of self-confidence when confronted with a caesarean section or a complex tear in an ordinal-ranked 0–5 scale. They graded the c-section with an average score of 4.41 (SD 0.65) and the complex tear with an average of 3.26 (SD 0.9), respectively, (*P* < 0.001). 

During their first perineal tear, 42 (91%) were supervised (95% CI: 79.2–97.6). About 33 (72%) did not conduct clinical followup after a tear (95% CI: 57.0–82.0), although 41 (89%) said “they did know the risk of urinary or faecal incontinence on the basis of whether the delivery is spontaneous or instrumental (95% CI: 79.2–97.6)”. Finally, 42 (91%) thought that it was necessary to receive more theoretical training (95% CI: 79.2–97.6), and 45 (98%) thought there was a need for a theoretical-practical course on pelvic floor anatomy and on the repair of its injuries (95% CI: 88.5–99.9). 

### 3.2. Discussion

This study provides information concerning how residents view their training in the repair of obstetric perineal trauma and looks at how they practice, how they are supervised, and how they followup patients. Before this study, there was a perceived deficit in the current training of residents in pelvic floor repair at the time of vaginal delivery as there are no formal, written educational objectives in the residency programme. It seemed important to objectively demonstrate this perceived deficit, so that this information might help revise what is currently being taught in resident training. The results indicate that actions such as practical workshops or the objective evaluation of skills should be carried out to reinforce residents' training in this area, in all teaching hospitals in Catalonia.

There are no differences in the assessments done by third and fourth year residents, and there are no discrepancies between second and third level hospitals. Neither were there differences whether the number of residents in the hospital was equal to, less than 2 or more than 2 or whether the number of deliveries per resident was more than 1000 or less (Table 3). 

We have no explanation for why no questionnaire was received from one of the 16 hospitals. 

The majority of residents think they do not have sufficient knowledge of pelvic floor anatomy. However, they feel confident in knowing when an episiotomy is required. This could be partly due to the fact that 80% of the participating hospitals use episiotomy in a restrictive manner.

We observed a clear difference in the self-confidence that residents show in the performance of a caesarean section when compared with the repair of a third or fourth degree perineal tear. On the one hand, the incidence of third degree tears in Catalonia is about 0-1% [18] underlining the high probability that many tears are underdiagnosed, although the cumulative incidence of AI postpartum in the same area is 4.5% (95% CI 3.1–5.9) [18]. On the other hand, according to an international study on cesarean rates worldwide, the reported incidence of caesarean section in Southern Europe is about 24% [19]. The rate in Spain was about 18% in 1999, approaching 21% in 2004 [20]. Since the incidence of c-section is much higher than that of third and fourth degree tears, not surprisingly residents feel far more confident in performing the c-section when confronted with the repair of a complex tear. This result is of high clinical significance. It is important to note, when explaining this result, that 70% of the residents have repaired less than 10 third or fourth degree tears, even though the number of caesarean sections carried out by each of them is far higher. 

Although residents claim that they know the consequences that a particular damage to the pelvic floor might entail for a patient, it is alarming that nearly 72% admit that they do not regularly followup women during the postpartum period. This fact suggests that even teachers and seniors have a limited understanding of the real problem [21]. In our region, it is common that puerperae make their postpartum check at one month after delivery with their midwife or gynaecologist in their corresponding primary care centre. Many hospitals do not yet have strong pelvic floor units. Consequently, the puerperae coming from these hospitals are still visited in their primary care centres at one month after their delivery, whatever their complications might have been. Thus, being aware of the implications of these injuries, it is of the utmost importance that a guideline is designed in all hospitals that ensures the followup of these patients. The followup is aimed at providing them with a good recovery from the morbidity, together with information and counselling for future pregnancies. 

Although the majority of the residents were supervised on their first repair, they believe that increased theoretical-practical training is still necessary. As recommended by other international societies [22], we strongly believe that it is important that part of the workload of subspecialists in urogynaecology at each hospital is devoted to active involvement in obstetrics. This may be appropriate in terms of preventing pelvic floor dysfunction on the labour ward through the education of residents and midwives, and by being present to help diagnose and treat anal sphincter injuries when they occur. Complementary to this, as shown by some authors, the implementation of tools that allow for the structured assessment of technical skills for the repair of fourth degree tears may be useful [23, 24]. We think that efforts should be made to regularly include such tools as a part of the residents' education programme in Catalonia. 

Not many surveys have been conducted which address this particular aspect of an obstetrics and gynaecology resident's education programme. Other surveys have shown that the urogynaecology training of general obstetrics and gynaecology residents should be revised and improved [25, 26], and some of the urogynaecology abilities queried in such surveys may be similar to an obstetric anal sphincter repair (i.e., posterior colporrhaphy, anal sphincteroplasty). Although most of these surveys have been conducted in English speaking countries, whose health care systems are different from that of Spain, their results are consistent with those of our own study, indicating that the Catalan residents' needs may be similar to those of residents in other countries. 

As the incidence and prevalence of faecal incontinence in Catalonia and Spain are similar and parallel to those published in the international literature [27], and, due to its cultural proximity, we believe that our results may also be representative of the situation in the rest of Spain. 

One of the limitations to this study was the sample size, which poses a problem regarding the statistic power needed to obtain statistically significant differences when crossing the affiliation data with the different items. In order to expand the sample size, it may be necessary to extend the survey to a national level, thereby allowing more significant conclusions to be drawn. Nonetheless, the population of Catalonia may be sufficiently representative of the rest of Spain to at least conclude that the lack of confidence when dealing with major perineal tears may be applicable nationwide. 

Furthermore, we should have taken certain measures to ensure greater participation in the questionnaire, since 64% participation may be below what is considered acceptable for a survey to support a valid study. 

Based on the results of our study, it appears that a thorough discussion and debate on residents' education and the prevention of pelvic floor dysfunctions caused by obstetric trauma should be undertaken by scientific societies in Spain. 

## 4. Conclusions

According to the results of the 46 questionnaires received, it can be concluded that there is a need to improve the training of residents in the management of perineal injuries during delivery and postpartum followup. This may be achieved by including theoretical and practical courses to reinforce pelvic anatomy and suture skills, and creating units to followup patients that are affected by complicated tears.

## Figures and Tables

**Table 1:** Demographics of participants.

	Total	**%**
Received questionnaires	46/72	64%
Represented teaching hospitals	15/16	94%
Third year residents	24/46	52%
Fourth year residents	22/46	48%
Female residents	37/46	80%
Male residents	9/46	20%

**Table 2:** Aggregate index rates calculated for each block of questions. Higher scores indicate better results.

	Anatomy questions	Episiotomy questions	Tear questions	Teaching questions
Valid participants	46	45	46	44
Lost participants	0	1	0	2
Mean score	3,17	4,29	3,19	10,79
Median's score	3,00	4,00	3,00	11,00
SD	1,48	0,84	0,96	1,68
Minimum score	1,00	2,00	1,00	6,00
Maximum score	6,00	5,00	5,00	14,00

**Table 3:** Mean scores per block. Higher scores indicate better results (mean ± SD). There were no significant differences in anatomy, knowledge of episiotomy, or knowledge of perineal tears between 3rd and 4th year residents, residents from different hospital levels, number of residents per hospital, or number of deliveries per resident. *Level III hospitals are referral hospitals.

	Year of residence		Hospital level*		Residents per hospital		Deliveries per resident	
	3rd	4th	II	III	1 or 2	>2	<1000	≥1000
Anatomy Questions	3.46 ± 1.61	2.86 ± 1.28	2.85 ± 1.57	3.30 ± 1.45	3.16 ± 1.63	3.10 ± 1.30	2.92 ± 1.32	3.48 ± 1.63
Episiotomy Questions	4.30 ± 0.76	4.27 ± 0.93	4.00 ± 1.08	4.41 ± 0.71	4.30 ± 0.91	4.30 ± 0.80	4.16 ± 0.99	4.45 ± 0.60
Tear questions	3.04 ± 1.04	3.36 ± 0.85	2.92 ± 1.04	3.30 ± 0.92	2.62 ± 0.57	3.19 ± 0.93	3.28 ± 0.98	3.09 ± 0.94
Teaching Questions	11.00 ± 2.02	10.57 ± 1.21	10.46 ± 2.02	10.93 ± 1.53	10.67 ± 1.71	10.90 ± 1.70	10.56 ± 1.85	11.04 ± 1.46

Total	21.86 ± 3.90	21.00 ± 2.49	20.23 ± 3.92	21.97 ± 2.87	20.75 ± 3.43	21.44 ± 2.81	20.78 ± 3.46	22.20 ± 2.95

**Table 4:** Questionnaire.

	Affiliation (mark with a cross)				
1	Year of residence	R3		R4	
2	Hospital	Level II		Level III	
3	Number of births/year (2006)
4	Number of residents/year
5	Gender	Male		Female	

	Evaluation on the knowledge of anatomy (mark with a cross)				

6	Do you think you have adequate knowledge of the pelvic floor anatomy?	YES		NO	
7	Do you know the name of the various muscles of the pelvic floor?	YES		NO	
8	Are you able to recognize the various muscles of the pelvic floor during digital vaginal examination?	YES		NO	
9	Are you able to identify the tendinous arc of the anus levator during digital vaginal examination?	YES		NO	
10	Can you identify the sciatica spines during digital vaginal examination?	YES		NO	
11	Do you know the path of the pudenda nerve, and are you able to inject the anaesthetics in it?	YES		NO	

	Evaluation on the knowledge of episiotomy (mark with a cross)				

12	Do you know when an episiotomy would be indicated?	YES		NO	
13	Are you familiar with the suture of the various types of episiotomy (medial, medial lateral)?	YES		NO	
14	Do you know when the medial episiotomy is counter indicated?	YES		NO	
15	Does your centre apply a selective policy for episiotomy?	YES		NO	
16	Do you think a selective policy for episiotomy is positive?	YES		NO	

	Evaluation on the knowledge of the perineal tears (mark with a cross or rate from 0 to 5)				

17	Do you know the definition of grades III and IV perineal tears?	YES		NO	

	Classification of perineal tears grade I: affects the vaginal mucosa and the connective tissue; grade II: affects the underlying muscles in addition; grade III: anal sphincter rupture; grade IV: affects the rectal mucosa				

18	Do you feel able to carry out a C-section? (rate from 0 to 5)
19	Do you feel able to repair a grade III or IV perineal tear? (rate from 0 to 5)
20	How many grade III or IV perineal tears have you repaired?	0–10	10–20		>20
21	Can you distinguish between a grade III and a grade IV perineal tear during the usual practice?	YES		NO	
22	Do you know more than one technique to repair an anal sphincter injury?	YES		NO	
23	Do you know when prophylactic antibiotics should be administered after a tear?	YES		NO	

	Evaluation on teaching (mark with a cross)				

24	Do you feel competent when repairing a perineal tear?	YES		NO	
25	Do you usually followup on a puerpera who has suffered a grade III or IV perineal tear after discharge (followup, pain level, sexual and anal dysfunctions)?	YES		NO	
26	Do you know if there is any difference in the risk of urinary and faecal incontinence after birth (due to mechanical lesion) depending on whether the birth is spontaneous or instrumental?	YES		NO	
27	Do you know if any of the instruments (spatules, forceps, vacuum pads) have higher associated risk or are their risks equivalent?	YES		NO	
28	Did you have a teaching assistant help during your suture in your first perineal tear repair?	YES		NO	
29	Did an assistant supervise/help you on subsequent occasions?	YES		NO	
30	Or did a major resident, in the absence of assistants?	YES		NO	
31	Do you think that you received adequate supervision when faced with a grade III or IV perineal tear?	YES		NO	
32	Do you think you can teach a minor resident to repair a grade III or IV perineal tear, in practice?	YES		NO	
33	Have you received formal training on pelvic anatomy or on the repair f perineal lacerations, within your training programme?	YES		NO	
34	Have you received theoretical training in any clinical session, videos, articles offered by any assistant?	YES		NO	
35	Have you read books, articles related to pelvic anatomy, perineal tears, episiotomy, surgical techniques for repairs, and so forth?	YES		NO	
36	Have you received any theoretical-practical training with corpses?	YES		NO	
37	Do you think you need to receive more supervision by an assistant or major resident to repair grade III or IV perineal tears?	YES		NO	
38	Do you think you need more theoretical training on it?	YES		NO	
39	Do you think a theoretical-practical course on the anatomy of he pelvic floor and the repair of its lesions would be useful?	YES		NO	
40	Would you give the same replies if you reread the questions after some minutes?	YES		NO	
